# Brain functional connectivity based on phase lag index of electroencephalography for automated diagnosis of schizophrenia using residual neural networks

**DOI:** 10.1002/acm2.14039

**Published:** 2023-06-06

**Authors:** Hasan Polat

**Affiliations:** ^1^ Department of Electrical and Energy Bingol University Bingöl Turkey

**Keywords:** deep learning, electroencephalogram, functional connectivity, phase lag index, ResNet, schizophrenia

## Abstract

The complexity of symptoms of schizophrenia (SZ) complicate traditional and effective diagnoses based on clinical signs. Moreover, clinical diagnosis of SZ is manual, time‐consuming, and error‐prone. Thus, there is a requirement to develop automated systems for timely and accurate diagnosis of SZ. This paper proposes an automated SZ diagnosis pipeline based on residual neural networks (ResNet). To exploit the superior image processing capabilities of the ResNet models, multi‐channel electroencephalogram (EEG) signals were converted into functional connectivity representations (FCRs). The functional connectivity of multiple regions in the cerebral cortex is critical for a better understanding of the mechanisms of SZ. In creating the FCR input images, the phase lag index (PLI) was calculated based on 16‐channel EEG signals from 45 SZ patients and 39 healthy control (HC) subjects to reduce and avoid the volume conduction effect. The experimental results showed that satisfactory classification performance (accuracy = 96.02%, specificity = 94.85%, sensitivity = 97.03%, precision = 95.70%, and F1‐score = 96.33%) was achieved by combining FCR inputs of beta oscillatory and the ResNet‐50 model. The statistical analyses also confirmed that there is a significant difference between SZ patients and HC subjects (*p* < 0.001, one‐way ANOVA). More specifically, the average connectivity strengths between nodes in the parietal cortex and those in the central, occipital, and temporal regions were significantly reduced in SZ patients compared to HC subjects. Overall results demonstrated that this paper not only provided an automated diagnostic model whose classification performance is superior to most previous studies but also valuable biomarkers for clinical use.

## 1 INTRODUCTION

Schizophrenia (SZ) is a common and severe neuropsychiatric disorder from which 1% of the worldwide population suffers.^1^ SZ causes some characteristic symptoms, including delusions, hallucinations, disorganized speech, abnormal memory, and reduced will.^2^ The negative symptoms broadly cause permanent disability and hamper daily routine. Furthermore, the mortality rate in SZ is two or three times higher than in the healthy population due to various diseases such as metabolic diseases, cardiovascular disorders, and infections.^3^ Typical symptoms usually first appear at the end of adolescence and the beginning of early adulthood.^4^ Although there is no permanent cure for SZ, the severe symptoms can be alleviated for many patients with appropriate treatment. Therefore, a timely and accurate diagnosis of SZ is crucial for starting treatment without delay and reducing the risk of pharmacological substance misuse.^5^

SZ diagnosis typically relies on interviews with patients and the existence or absence of representative behavioral symptoms.^6^ The complexity of SZ symptoms hamper traditional and effective diagnoses based on clinical signs.^4^, ^7^ Moreover, the fact that psychotic symptoms such as hallucinations and delusions are not distinctive to SZ but might be present in other neurodegenerative disorders increases the need for support systems for accurate diagnosis. In this context, sophisticated brain imaging techniques, such as computed tomography (CT), magnetic resonance imaging (MRI), electroencephalogram (EEG), and positron emission tomography (PET), have become promising diagnostic tools for neurology and mental health professionals.^5^ Compared to other imaging methods, the low‐cost, high temporal resolution of non‐invasive EEG signals and the ease of use even in remote locations with minimal instruments make it a viable option for examining the relationship between oscillatory brain activity and cognitive dysfunction.^5^, ^8^

EEG provides a straightforward measurement of brain electrical activity along the scalp and is a well‐known neurophysiological SZ biomarker. It is a rich source of information that allows quantitative assessment of cognitive function and mental health.^9^ In EEG signals, increasing/decreasing band power for specific frequencies, complexity, and synchrony are the main arguments for researchers. However, SZ diagnosis based on visual EEG analysis is time‐consuming, requires specialized training, and is error‐prone.^10^ Machine learning‐based diagnostic systems may provide a critical path for a better assessment of EEG.^4^ Automatic diagnosis of SZ using EEG signals commonly consists of three core components: Preprocessing steps (artifact suppression and segmentation), feature extraction, and discrimination between patients with SZ and healthy control (HC) subjects.^11^ However, the problem of identifying SZ abnormalities from EEG signals to aid clinical diagnosis and treatment remains open. One of the reasons for this problem is that most of the proposed diagnostic pipeline is based on hand‐crafted methods consisting of feature extraction and conventional classifiers. In the feature extraction phase of these studies, a set of discriminative hand‐crafted features is extracted from the EEG signals. In this context, a variety of linear and nonlinear features have been used for automated SZ diagnosis, including entropic complexity,^9^, ^12^, ^13^ statistical approaches,^14^, ^15^ spectral features,^16^, ^17^ empirical mode decomposition,^18^ and time‐frequency domain features.^19^, ^20^ The feature engineering before classifier causes the overall flow to be cumbersome and time‐consuming.^21^ Furthermore, it directly affects model performance.

After feature extraction, an appropriate diagnostic model for the neuropsychiatric abnormality is developed using one of several classification methods. Previous studies have used various classification methods to diagnose SZ based on EEG signals, including support vector machine (SVM),^9^, ^14^, ^20^ random forest (RF),^9^, ^17^ linear discriminant analysis (LDA),^12^ adaptive boosting (Adaboost),^15^ naive Bayes (NB),^14^, ^15^ and decision tree (DT).^14^ Furthermore, many studies have achieved the optimal diagnostic pipeline by experimenting with different types of well‐known classifiers in addition to a proposed novel feature extraction method.^1^, ^5^, ^9^, ^14^, ^15^

Deep learning (DL) has exhibited an unprecedented level of accuracy in various biomedical task. It bypasses hand‐crafted feature engineering and automatically operates the feature extraction and classification steps.^6^, ^11^, ^22^ However, the performance of the DL is directly related to the formulation of the inputs. For the diagnosis of SZ, several researchers have proposed various DL models, and the inputs have been fed either directly raw EEG signals or their reconstructed multidimensional representations. Chu et al.^23^ aimed to increase the performance in SZ diagnosis by making various modifications to the DL pipeline. The proposed framework demonstrated a consistent advantage by replacing the softmax layer with RF conventional classifier. The modified DL model achieved an overall classification accuracy of 92.5%. Oh et al.^6^ proposed a computerized detection system for the diagnosis of SZ using an eleven‐layered convolutional neural network (CNN). For input formulation, they used time‐domain representation to exploit the proposed CNN model. Their model achieved an accuracy of 98.07% and 81.26% for subject‐based testing and non‐subject‐based testing, respectively. Phang et al.^24^ explored the combination of various connectivity features consisting of time and frequency domain metrics of effective connectivity to detect SZ abnormalities. They converted one‐dimensional EEG signals to multi‐domain connectivity maps and fed them into multi connectome CNN classifier. Shalbaf et al.^25^ proposed an optimal SZ diagnostic model by testing multiple popular CNN architectures. First, EEG signals were converted into images by applying a time‐frequency approach called the scalogram method. For automated feature extraction, they employed the four popular pre‐trained CNNs: AlexNet,^26^ ResNet‐18,^27^ VGG‐19,^28^ and Inception‐v3.^29^ The deep features of these models were used as inputs of the SVM classifier. The results demonstrated that ResNet18+SVM was the most effective combination for automated SZ diagnosis. Calhas et al.^30^ proposed a pairwise distance learning technique for automated SZ detection relying on the spectral properties of the one‐dimensional EEG series. They exploited time‐frequency representation to indicate discriminative feature space from pairwise combinations of observations per channel. The SZ diagnostic model was tested on reference clinical trial data under the resting‐state paradigm and achieved a classification accuracy of 95%.

The previous works mainly focus on single‐channel EEG signals to describe the pattern of brain activity caused by neurological abnormalities and ignore the functional connectivity between brain regions.^31^ Functional connectivity of multiple regions in the cerebral cortex is crucial to better understanding the mechanisms of various neurological diseases.^32^ It can provide robust multidimensional representations of EEG in detecting SZ abnormalities. Therefore, this paper proposes converting the multi‐channel EEG recording into functional connectivity representation (FCR) and feeding them into a residual blocks‐based convolutional neural network (ResNet) for automated SZ detection. Here, the phase lag index (PLI) is calculated based on the multi‐channel EEG signals to create the FCR as input. PLI allows for the quantification of phase synchronization. It has a significant advantage due to the insensitivity to volume conduction effect.^33^ This paper focuses on brain connectivity dynamics for specific frequency rhythms. PLI‐based brain connectivity maps are constructed separately for delta, theta, alpha, beta, and gamma rhythms, and the robustness of each frequency range in diagnosing SZ is evaluated. Additionally, the connectivity strengths of different regions along the cerebral cortex of HC subjects and SZ patients were explored.

The rest of the paper is structured as follows: Section 2 describes the materials and methods, including the EEG database, preprocessing phase, creation of FCR inputs, and residual neural networks. Section 3 provides experimental results of the proposed frameworks. Section 4 discusses the contributions, superiorities, and limitations of the proposed model, and the last section concludes the proposed work.

## 2 MATERIAL AND METHODS

**FIGURE 1:**
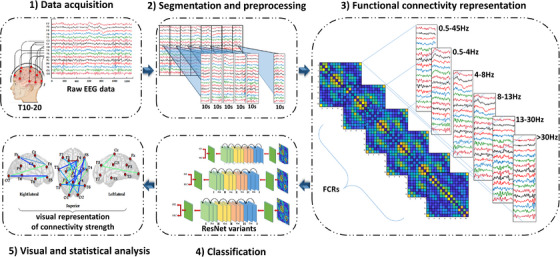
Overview of the proposed pipeline for the diagnosis and evaluation of SZ.

The proposed pipeline involves multi‐channel EEG recordings, artifact handling or filtering, segmentation process, the FCR input formulation based on PLI method, technical detail of residual neural networks, hyperparameter optimization, and evaluation metrics. To clarify the methodology of this paper, Figure 1 illustrates an overview of the proposed SZ diagnosis framework.

### 2.1 Data acquisition

The proposed methodology for the automated diagnosis system was applied to a publicly available EEG dataset.^9^, ^30^, ^34^ The EEG recordings belong to two groups. The first one consists of 45 adolescent patients (10−14 years old) with SZ. All patients (including childhood SZ, schizotypal, and schizoaffective disorders) were diagnosed at the Mental Health Research Center (MHRC) according to different SZ diagnostic criteria. The second group consists of 39 healthy adolescents (11−13 years old). For all participants, EEG recordings were obtained with 16 electrodes (O1, O2, P3, P4, Pz, T5, T6, C3, C4, Cz, T3, T4, F3, F4, F7, F8) in the standard 10 20 international placement referenced to linked earlobe electrode and impedance for all electrodes were checked to remain below 10 kΩ.

**FIGURE 2:**
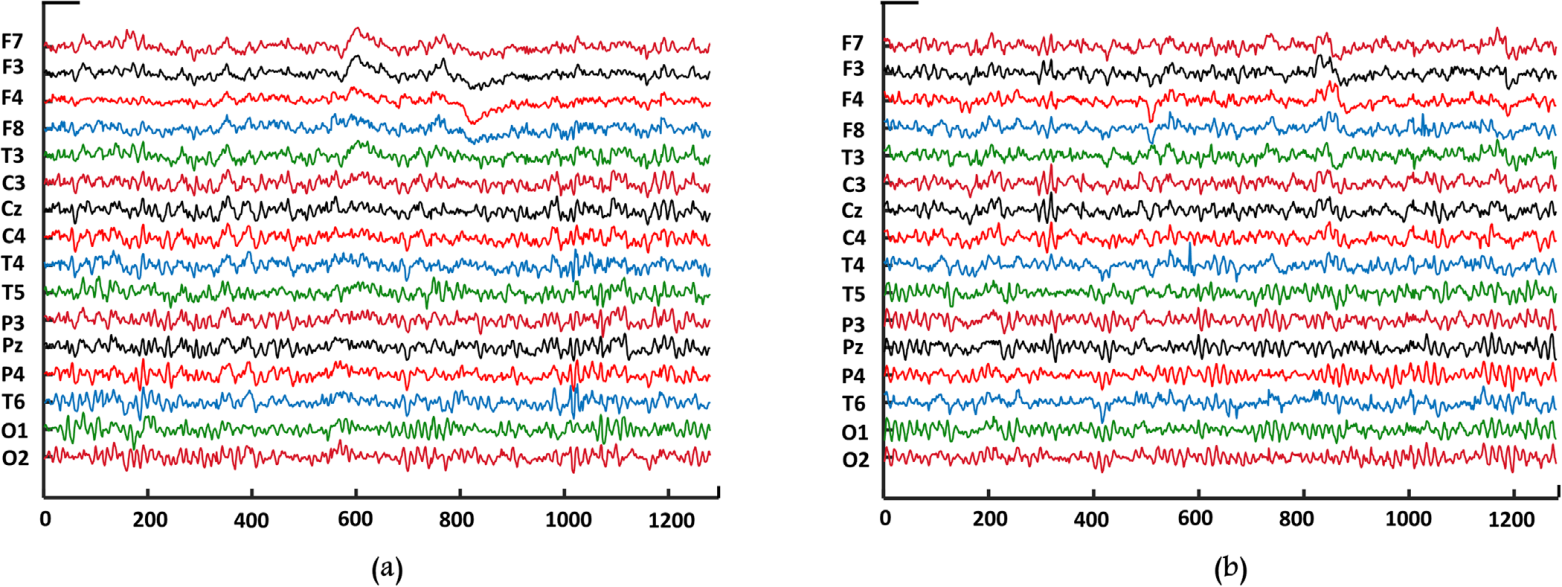
Samples of multi‐channel EEG recordings in the resting state with eyes closed (a) HC (b) SZ.

Artifacts produced by eye blinks and eye movements were manually subtracted based on the assessment of two experts. Therefore, the data are made available free of artifacts. During the recordings, patients were in resting condition, with eyes closed. The signal was recorded for 1 min with a sampling rate of 128 Hz. The length of each EEG segment after artifacts suppression was 7680 points. Figure 2 shows exemplary EEG recordings with a duration of 10 s from SZ patients and HC subjects.

### 2.2 Data segmentation and preprocessing

EEG signals are non‐stationary and reflect the complex dynamics of the brain. However, EEG signals used for computer‐based models are assumed to be stationary in the relevant period. To overcome this conflict, EEG signals are usually divided into a series of short windows. EEG characteristics are assumed to be approximately stationary within each window.^1^ The literature reports that various window sizes have been used in computer‐assisted SZ detection, including 2 s,^1^ 5 s,^12^ 10 s,^34^ 25 s,^5^ and longer periods.

In this study, the EEG data were segmented by applying a moving window of 10 s with no overlapping. The segmentation process provided 6 × 16 = 96 EEG epochs for each participant. In this way, for all 84 participants, a total of 84 × 96 = 8064 EEG segments (1 × 1280 data points) were created. All the EEG samples of each participant were band‐pass filtered into the five well‐known frequency rhythms: delta (0.5−4 Hz), theta (4−8 Hz), alpha (8−13 Hz), beta (13−30 Hz), and gamma‐range (> 30 Hz).

### 2.3 Brain functional connectivity based on phase lag index

Functional connectivity features of the brain have great potential for investigating abnormalities in neurological diseases. In this study, the PLI technique was used to assess functional connectivity between EEG channels and to generate FCR patterns. The PLI is an effective tool for analyzing the functional connectivity of the brain.^35^, ^36^ It provides the degree of pairwise connectivity. PLI is a metric of the asymmetry of the distribution of instantaneous phase differences between two EEG channels.^31^ Stam et al.^33^ describe the formula of the PLI value between two signals as

(1) $$PLI\;=\left|\frac{1}{T}\sum_{t=1}^{T}sgn\left(\Delta \varphi \left(t\right)\right)\right|$$

**FIGURE 3:**
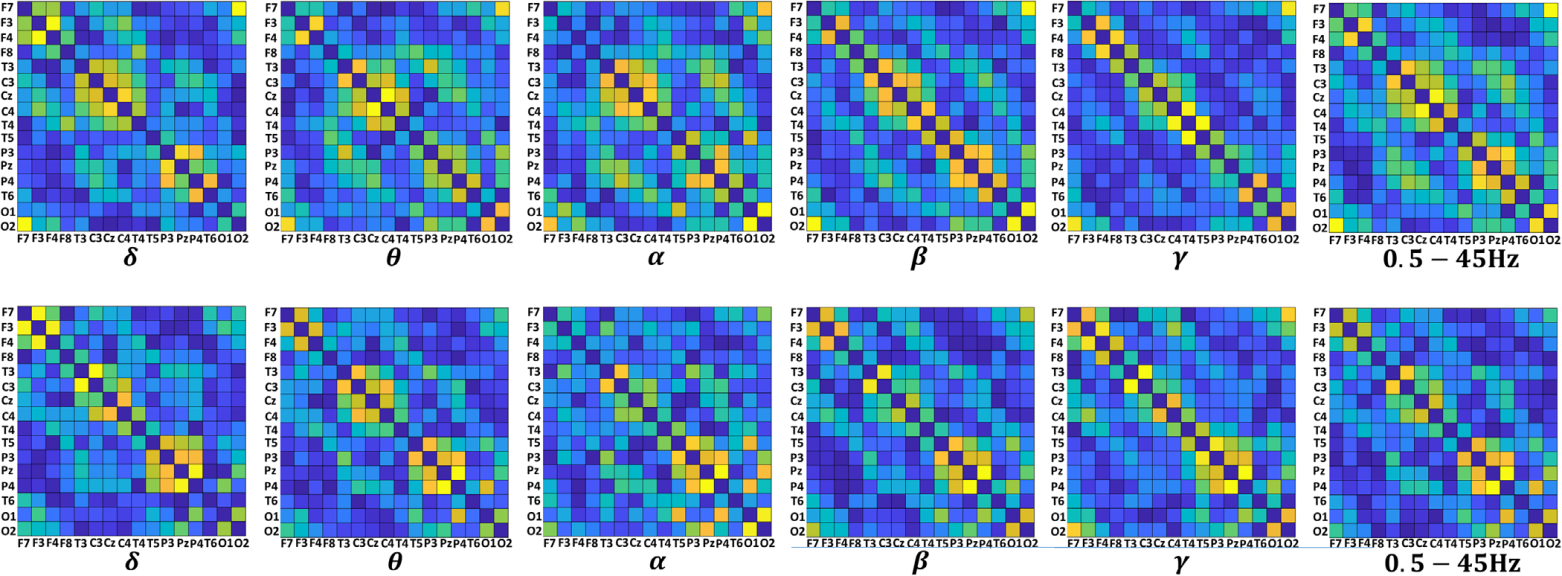
Visualization of symmetric connectivity matrices for obtaining FCR inputs. The first row shows examples from SZ and the second row shows examples from HC data. The columns represent the frequency ranges delta, theta, alpha, beta, gamma, and 0.5–45 Hz, respectively.

Here, $\Delta \varphi \;\left(t\right)=\varphi_{1}\;\left(t\right)-\varphi_{2}\left(t\right)$ is the phase differences between two EEG segments, sgn() is the signum function, t∈{1,2,3,…,T} denotes discrete time values, $\varphi_{1}\left(t\right)$ and $\varphi_{2}\left(t\right)$ are the phase components of two EEG segments. The range of Δφ(t) is determined from two complex signals converted using the Hilbert transform. PLI quantifies the pairwise connectivity strength with a range from 0 to 1. More specifically, PLI = 0 indicates no coupling, while PLI = 1 indicates perfect phase coupling for two EEG segments.^31^, ^33^, ^35^ After the PLI analysis, 16 × 16 symmetric connectivity matrices are obtained for each 16‐channel EEG recording. The connectivity matrices represented are employed to obtain the FCR as the input of the proposed DL pipeline. In other words, FCR is a type of topographic visualization of symmetric connectivity matrices. Figure 3 demonstrates sample FCR inputs for each frequency band related to HC subjects and SZ patients.

In this study, multi‐channel EEG epochs consisting of 16 × 1280 samples were transformed into topographical representations of symmetrical connectivity matrices as 342 × 342 pixel images by using the PLI method. In order to reduce the processing load of the ResNet algorithm and generate an appropriate input, the FCR images were resized into 224 × 224 pixels.

### 2.4 Residual neural networks

After preparing the input dataset from one‐dimensional time series, the most crucial step is to design the proper DL architecture to discriminate SZ patients from HC subjects. CNN, one of the DL models, has several advantages over other deep architectures, including being more similar to the human visual processing system and having a structure optimized for processing 2D or 3D images.^37^ Various CNN architectures have been proposed in the previous works. The proposed CNN architectures have been progressively deeper for higher performance. However, the deeper neural networks are more difficult to train due to the problem of vanishing and exploding gradients.^27^, ^37^, ^38^, ^39^ In this study, ResNet architecture, which overcomes these problems, is used for feature extraction and classification processes.

**FIGURE 4:**
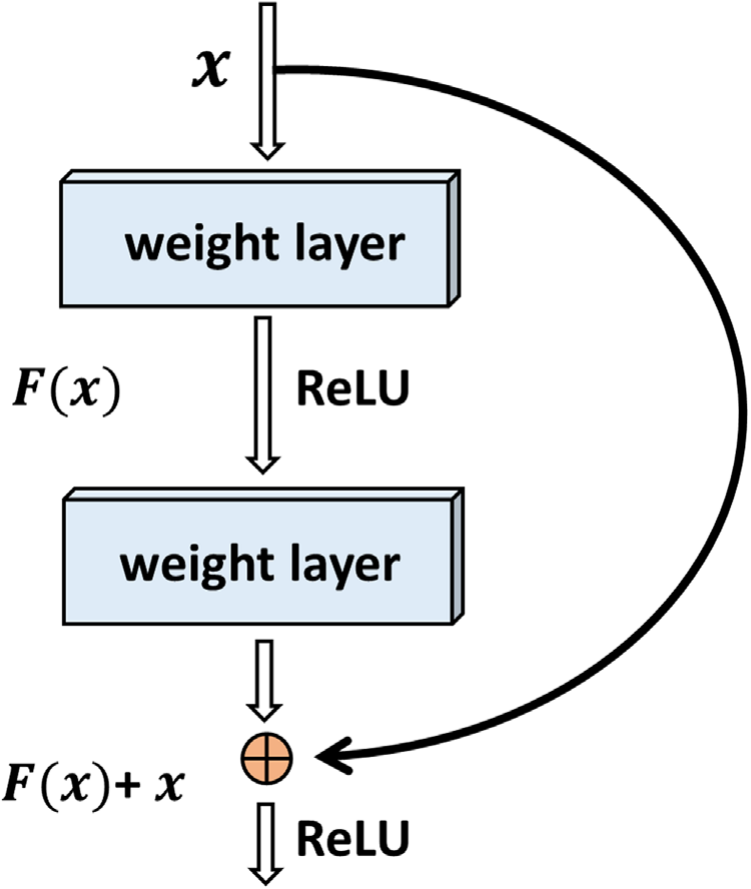
A residual block.

ResNet is a conventional feedforward end‐to‐end network based on the residual block or skip connection.^27^ Output formulations of residual blocks (F(x) + x) can be realized by feedforward neural networks with shortcut connections. More precisely, the shortcut connections achieve identity mapping, and their outputs are added to the outputs of the stacked layers.^27^ This implementation adds neither extra parameters nor computational complexity. Because there is no increase in the number of learnable parameters, the network is advantageous in that it can still be trained end‐to‐end with backpropagation. More specifically, the novelty of the ResNet idea is to design deeper networks without incurring the problem of vanishing and exploding gradients. Hence, residual networks can achieve a high level of accuracy from considerably increased depth.^27^, ^37^, ^40^ Figure 4 shows a basic diagram of the residual block.

There are several variants of ResNet with different numbers of layers, for example, ResNet‐18, ResNet‐34, ResNet‐50, and ResNet‐101. Although different ResNet models have a different number of layers, the overall pipelines of these architectures share a common form. ResNet essentially has five convolution blocks to obtain output spatial resolution. The number of convolution layers in each block varies by architecture. This paper investigated the effectiveness of the ResNet‐18, ResNet‐50, and ResNet‐101 models and determined the most robust variant of residual networks for automated SZ diagnosis. Table 1 shows the proposed ResNet18, ResNet50, and ResNet101 architectures for ImageNet.

**TABLE 1:** ResNet‐18, ResNet‐50, and ResNet‐101 architectures developed for ImageNet.

Layers	Output size	ResNet‐18	ResNet‐50	ResNet‐101
Conv1	112 × 112	7×7,64 stride 2	7×7,64, stride 2	7×7,64, stride 2
Conv2_x	56 × 56	3×3maxpool stride 2	3×3maxpool stride 2	3×3maxpool stride 2
		$\left[\begin{array}{c} 3\times 3,\;64\\ 3\times 3,\;64 \end{array}\right]\times 2$	$\left[\begin{array}{c} 1\times 1,\;64\\ 3\times 3,\;64\\ 1\times 1,\;256 \end{array}\right]\times 3$	$\left[\begin{array}{c} 1\times 1,\;64\\ 3\times 3,\;64\\ 1\times 1,\;256 \end{array}\right]\times 3$
Conv3_x	28 × 28	$\left[\begin{array}{c} 3\times 3,\;128\\ 3\times 3,\;128 \end{array}\right]\times 2$	$\left[\begin{array}{c} 1\times 1,\;128\\ 3\times 3,\;128\\ 1\times 1,\;512 \end{array}\right]\times 4$	$\left[\begin{array}{c} 1\times 1,\;128\\ 3\times 3,\;128\\ 1\times 1,\;512 \end{array}\right]\times 4$
Conv4_x	14 × 14	$\left[\begin{array}{c} 3\times 3,\;256\\ 3\times 3,\;256 \end{array}\right]\times 2$	$\left[\begin{array}{c} 1\times 1,\;256\\ 3\times 3,\;256\\ 1\times 1,\;1024 \end{array}\right]\times 6$	$\left[\begin{array}{c} 1\times 1,\;256\\ 3\times 3,\;256\\ 1\times 1,\;1024 \end{array}\right]\times 23$
Conv5_x	7 × 7	$\left[\begin{array}{c} 3\times 3,\;512\\ 3\times 3,\;512 \end{array}\right]\times 2$	$\left[\begin{array}{c} 1\times 1,\;512\\ 3\times 3,\;512\\ 1\times 1,\;2048 \end{array}\right]\times 3$	$\left[\begin{array}{c} 1\times 1,\;512\\ 3\times 3,\;512\\ 1\times 1,\;2048 \end{array}\right]\times 3$
	1 × 1	Average pool, 100‐d fully connected layer, softmax		
Size		1.8 M	3.8 M	7.6 M

*Note*: Here, the building blocks are shown in brackets with the number of blocks stacked.^27^

### 2.5 Hyperparameters

The implementation of the proposed SZ diagnosis model requires the choice of some design parameters. In this study, the hyperparameters for the residual networks were tuned based on empirical trials. The network was trained using an adaptive moment estimation (Adam) optimizer. The initial learning rate was set to 0.001 and then decreased by 0.4 after every four epochs. Hence, the network did not get stuck at the local minimum. The mini‐batch size was set to 32, meaning that 32 samples (FCR patterns) were processed per iteration. The training session was terminated after 15 epochs. Furthermore, the early stopping method was employed to minimize the risk of overfitting.

**FIGURE 5:**
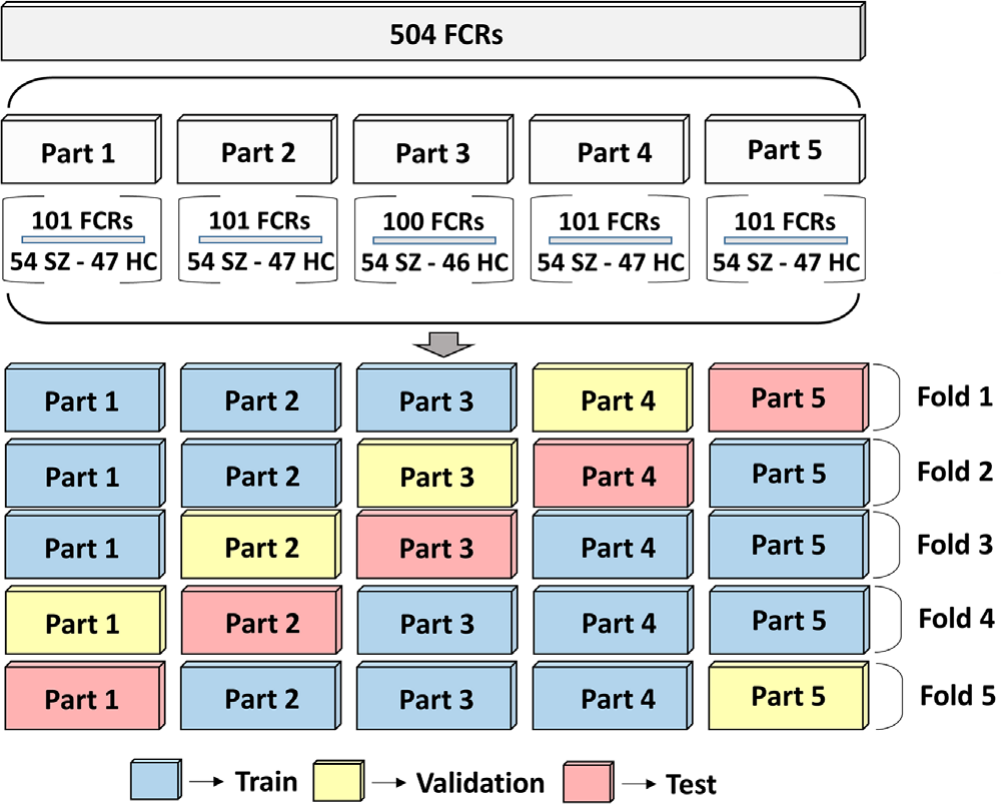
Schematic representation of the five‐fold cross‐validation technique.

The dataset, consisting of 504 FCR images for each specific frequency, is divided into three sets for training, validation, and testing, with ratios of 0.6, 0.2, and 0.2, respectively. To ensure reliable generalization, five‐fold cross‐validation was used. More specifically, the 504 FCR samples for a sub‐band (270 for the SZ patients and 234 for the HC subjects) were partitioned into five parts. In the partition process, it was tried to ensure that EEG segments of the same subjects were not included in different parts. In each classification fold, three of the parts were used for training, one for validation, and the rest for testing the model. At the end of this process, the data assigned for training, validation, and testing corresponds to 60%, 20%, and 20% of the whole data, respectively. Figure 5 illustrates the five‐fold cross‐validation process.

### 2.6 Evaluation metrics

To further analyze whether ResNet variants, it is crucial to select appropriate metrics. The ideal metrics should heavily focus on the ability of the proposed diagnostic model to detect the FCR patterns containing SZ abnormalities. In this study, the classification performance was quantified using accuracy, sensitivity (recall), specificity, precision, and F1‐score metrics.

(2) $$Accuracy\;=\frac{TP+TN}{TP+FP+TN+FN}$$

(3) $$Sensitivity\left(Recall\right)\;=\frac{TP}{TP+FN}$$

(4) $$Specificity\;=\frac{TN}{TN+FP}$$

(5) $$Precision\;=\frac{TP}{TP+TN}$$

(6) $$F1-score\;=\;2\times \frac{Precision\times Recall}{Precision+Recall}$$

where

TP (true positive): the number of correctly identified SZ segments.

TN (true negative): the number of correctly identified healthy control segments.

FP (false positive): the number of healthy segments misclassified as SZ segment.

FN (false negative): the number of SZ segments misclassified as healthy control segments.

## 3 RESULTS

This section presents the performance of different ResNet architectures that process FCR representations as input to discriminate SZ patients from HC subjects. Apart from the classification processes, a one‐way analysis of variance (ANOVA) was also performed to explore whether there were electrode pairs with significant differences between groups. In this study, all experiments were run on a desktop with having 2.7‐GHz Intel dual‐core i7 processor and NVIDIA GeForce ROG‐STRIX 256 bit graphics cards. The successive processing steps were implemented in a Matlab software environment, including EEG segmentation, filtering, PLI‐based connectivity representation, the application of ResNet variants, and a one‐way ANOVA.

### 3.1 Discriminating between HC subjects and SZ patients

The proposed diagnostic frameworks are performed separately for six specific frequency bands to achieve satisfactory discrimination between SZ patients and HC subjects. This strategy has a great potential to identify subband functional connectivity patterns that reflect SZ anomalies and provide the best input quality to the DL pipeline. Furthermore, it can determine which of the popular ResNet architectures better detect patterns of neuropsychiatric disorders from FCR images. Table 2 shows the overall classification performance (accuracy, specificity, precision, sensitivity, and F1‐score) for each ResNet variant and frequency band after the five‐fold cross‐validation.

**TABLE 2:** Overall results of classification performance based on three ResNet variants

Model	Subband	Acc.	Spe.	Sen.	Prec.	F1
	Delta	76,57	74,34	78,51	78,10	78,20
	Theta	81,54	81,63	81,48	83,80	82,50
ResNet‐18	Alpha	83,13	80,78	85,18	83,82	84,33
	**Beta**	**93,26**	**89,34**	**96,66**	**91,48**	**93,92**
	Gamma	86,11	83,74	88,14	86,58	87,18
	0.5‐45 Hz	90,87	90,15	91,48	91,51	91,44
	Delta	76,38	82,04	71,48	82,72	76,24
	Theta	82,14	85,05	79,62	87,34	82,69
ResNet‐50	Alpha	84,30	88,45	80,74	88,83	84,35
	**Beta**	**96,02**	**94,85**	**97,03**	**95,70**	**96,33**
	Gamma	89,48	87,63	91,11	89,99	90,31
	0.5‐45 Hz	91,67	94,04	89,62	94,69	92,03
	Delta	75,40	75,65	75,18	78,36	76,58
	Theta	81,14	76,91	84,81	81,49	82,86
	Alpha	76,98	70,94	82,22	78,91	79,68
ResNet‐101	Beta	91,07	87,60	94,07	90,39	91,90
	Gamma	88,29	88,43	88,14	90,08	88,70
	**0.5–45** **Hz**	**91,26**	**90,59**	**91,85**	**91,82**	**91,81**

*Note*: Bold values indicate concerning the method with the best performance.

Acc, Accuracy; Spe, Specificity; Sen, Sensitivity; Prec, Precision; F1, F1‐score.

Table 2 shows that the ResNet‐50 architecture performs significantly better than other variants in classifying SZ and HC functional connectivity patterns across almost all subband sets. After training, inferences revealed strong classification performance, especially for beta and 0.5–45 Hz functional connectivity features. Overall, the ResNet‐50 model achieved an accuracy of 96.02% and 91.67% for beta and 0.5–45 Hz frequency rhythms, respectively. Furthermore, it achieved specificity and sensitivity of 94.85% and 97.03% on the beta, as well as 94.04% and 89.62% on the 05−45 Hz frequency band, respectively. The sensitivity and specificity are crucial metrics due to the slightly imbalanced number of the FCR images assigned to the two classes in the test sets (54 SZ and 46/47 HC samples in each fold). The closeness of both metric values ​​indicates that the proposed classifier makes a good generalization between the two classes.

The ResNet‐18 and ResNet‐101 models achieved an accuracy of 90.87% and 91.07% for beta, as well as 90.67% and 91.26% for the 0.5–45 Hz frequency band, respectively. It has been reported that the sensitivity and specificity metrics are consistent for both models. In this context, using the beta functional connectivity patterns achieved a sensitivity of 96.66% on ResNet‐18 and 94.07% on ResNet‐101. The specificity performances obtained after five‐fold cross‐validation were 89.34% and 87.60% for ResNet‐18 and ResNet‐50, respectively.

**FIGURE 6:**
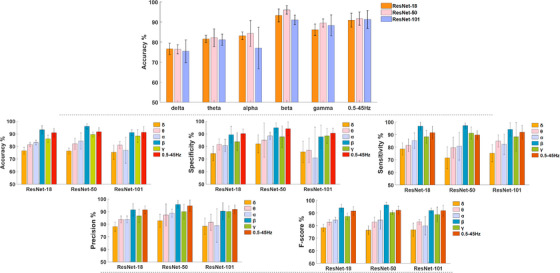
Bar graphs of the average and standard deviation of obtained results. The upper part compares the performance of the ResNet variants in different frequency bands, while the lower part shows the variation of the performance of each architecture across the subbands.

The precision performance of the discrimination between SZ patients and HC subjects examines whether the correctly classified samples of SZ patients are actual SZ patients and whether the rest are HC subjects incorrectly labeled as SZ. The F1‐score represents the average of the precision and sensitivity (recall). The highest precision was 95.70% for the beta oscillations and the ResNet‐50. For all variants of ResNet, the precision performance ranged from 91.48% to 95.70%. The ResNet‐50 model based on beta functional connectivity patterns achieved the highest value of F1‐score as 96.33%, followed by ResNet‐18 (93.92%), and ResNet‐101 (91.90%). On the other hand, the worst classification results for F1‐score for all variants of ResNet were obtained for delta frequency. To further evaluate the impact of each frequency band and ResNet variant on classification performance, Figure 6 comparatively shows the bar graphs of the mean and standard deviation of obtained results in the five‐fold training phase.

As shown in Figure 6, the beta functional connectivity patterns provided the best input quality for the DL networks. The classification accuracy achieved by the three different ResNet variants ranged from 91.07% to 96.02%. Figure 5 also shows that the accuracy performance of each classification fold was most regular for the beta frequency. The standard deviations for ResNet‐18, ResNet‐50, and ResNet101 were ±3.23%, ±2.11%, and ±2.32%, respectively. ResNet variants for 05−45 Hz FCR inputs showed the ability to discriminate SZ patients from HC subjects with similar accuracy. More specifically, the classification accuracy ranged from 90.87% to 91.67% for the 05−45 Hz band. On the other hand, the proposed residual networks based on the alpha functional connectivity features showed the highest variance during the five‐fold cross‐validation. After the five‐fold training, the results revealed that the highest standard deviation was ±10.33% for the ResNet‐101 model. Overall, the results revealed that higher frequency connectivity features, except for gamma rhythms, increased classification accuracy and better reflected the discriminative features of SZ anomalies

As shown in Figure 5, the beta functional connectivity patterns provided the best input quality for the DL networks. The classification accuracy achieved by the three different ResNet variants ranged from 91.07% to 96.02%. Figure 6 also shows that the accuracy performance of each classification fold was most regular for the beta frequency. The standard deviations for ResNet‐18, ResNet‐50, and ResNet101 were ±3.23%, ±2.11%, and ±2.32%, respectively. ResNet variants for 05−45 Hz FCR inputs showed the ability to discriminate SZ patients from HC subjects with similar accuracy. More specifically, the classification accuracy ranged from 90.87% to 91.67% for the 05−45 Hz band. On the other hand, the proposed residual networks based on the alpha functional connectivity features showed the highest variance during the five‐fold cross‐validation. After the five‐fold training, the results revealed that the highest standard deviation was ±10.33% for the ResNet‐101 model. Overall, the results revealed that higher frequency connectivity features, except for gamma rhythms, increased classification accuracy and better reflected the discriminative features of SZ anomalies.

**FIGURE 7:**
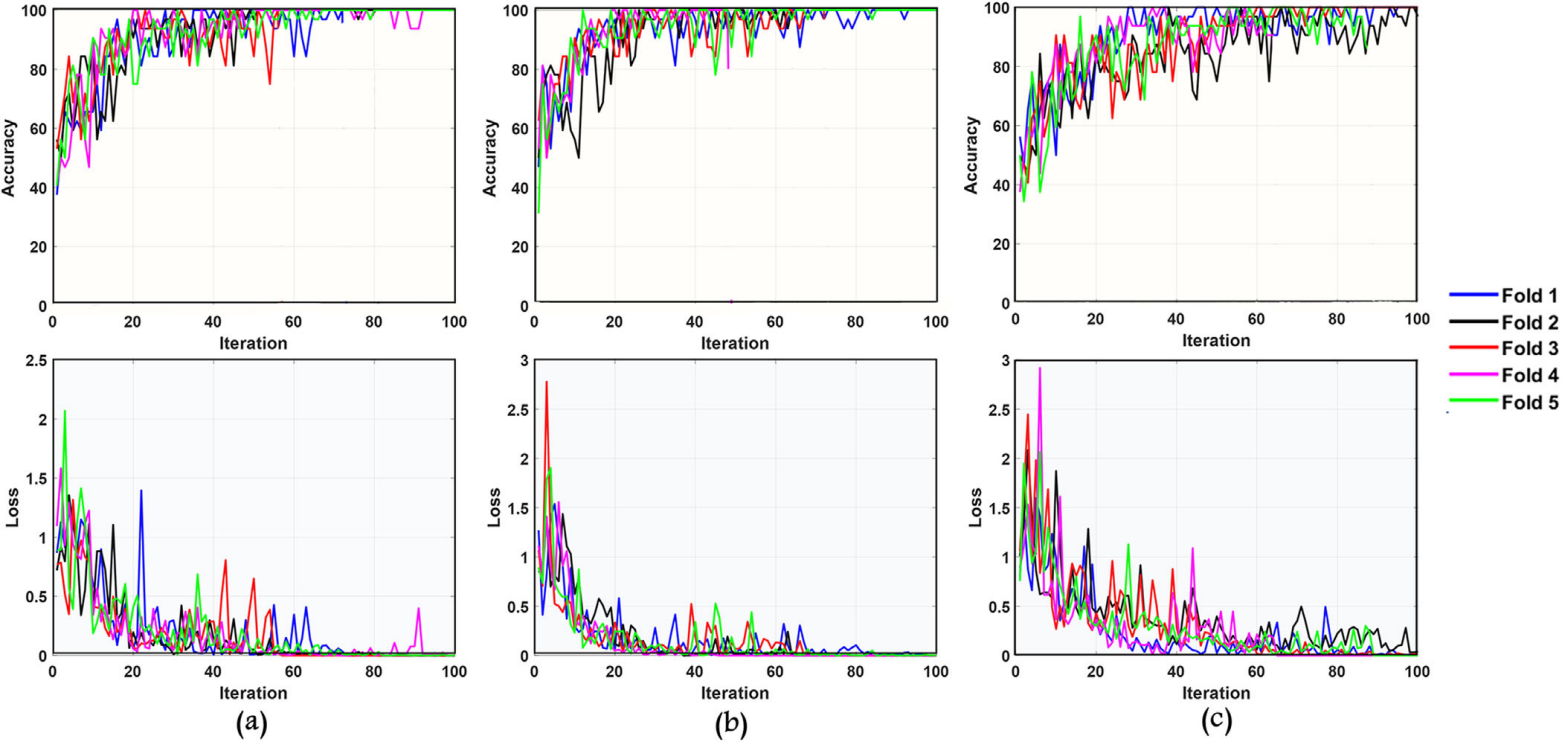
Loss and accuracy plots through the five‐fold training phase for different ResNet variants. (a): ResNet‐18, (b): ResNet‐50, (c): ResNet‐101.

ResNet‐50 performed the best performance in terms of accuracy but has a higher number of parameters than ResNet‐18. While ResNet‐50 completed the entire training process in 6.41 min with approximately 22.4 M parameters, ResNet‐18 completed the training process in 1.45 min with approximately 11.2 M parameters. Therefore, the classification pipeline based on ResNet‐18 has competitive performance in terms of the trade‐off. On the other hand, ResNet‐101 lagged behind the other two architectures in terms of performance. Figure 7 illustrates training performance and loss for different ResNet variants.

### 3.2 SZ effect on pairwise connections

After classifying the standard oscillatory components (δ,θ,α,β, andγ) of the EEG data with different ResNet variants, the obtained result demonstrated that the functional connectivity features of the beta band are the most efficient patterns for evaluating SZ abnormalities. To further analyze the effects of SZ on the brain, the degrees of pairwise coupling strengths along the cortical surface were estimated in terms of phase lag index. In this context, this paper attempted to determine whether the SZ and HC classes showed a statistically significant difference for each pair of EEG channels by performing a one‐way ANOVA test. Here, the significance level was taken into account in two different classes 0.001⩽*p*<0.05 and *p*<0.001. Functional connectivity features between EEG channels for SZ patients and HC subjects were evaluated separately at both levels of significance. Table 3 shows the statistical comparison between SZ patients and HC subjects using functional connectivity measurements based on PLI at a level of significance of 0.001⩽*p*<0.05.

**TABLE 3:** The result of statistical comparison according to significant differences between the patient and healthy groups according to significant differences level of 0.001 ⩽*p*< 0.05

		Connectivity strength (mean ± std)							Connectivity strength (mean ± std)				
Channels		SZ		HC		*p*	Channels		SZ		HC		*p*
F7	C4	**0.179**	±0.09	0.158	±0.10	0.008	F8	T4	**0.267**	±0.10	0.246	±0.08	0.008
F3	F4	**0.669**	±0.00	0.648	±0.08	0.005	F8	T5	**0.347**	±0.10	0.324	±0.09	0.005
F3	Cz	**0.310**	±0.09	0.284	±0.11	0.002	F8	O1	0.102	±0.08	**0.121**	±0.08	0.008
F3	P4	0.132	±0.09	**0.149**	±0.09	0.041	F8	O2	0.098	±0.06	**0.113**	±0.07	0.007
F3	O1	**0.318**	±0.08	0.300	±0.09	0.018	T3	O2	**0.148**	±0.09	0.128	±0.08	0.005
F4	T3	0.106	±0.07	**0.125**	±0.08	0.004	C4	Pz	0.108	±0.09	**0.132**	±0.09	0.002
F4	C3	0.190	±0.10	**0.210**	±0.12	0.026	T4	P3	**0.126**	±0.10	0.101	±0.08	0.001
F4	Cz	0.106	±0.09	**0.123**	±0.08	0.029	T4	O1	**0.321**	±0.09	0.300	±0.10	0.009
F4	Pz	0.085	±0.07	**0.098**	±0.07	0.028	T5	P4	0.325	±0.11	**0.347**	±0.11	0.025
F4	O2	**0.322**	±0.08	0.306	±0.09	0.035	T5	T6	0.164	±0.10	**0.182**	±0.09	0.036

*Note*: Bold fonts indicate higher connectivity strength.

Abbreviations: HC, Healthy control; *p*, *p‐*value; SZ, Sczofrenia.

As shown in Table 3, SZ patients showed decreased functional connectivity from occipital and parietal channels to the frontal channels (P4‐F3, Pz‐F4, O2‐F8, and O1‐F8) than did HC subjects. Furthermore, SZ patients exhibited lower functional connectivity from the central and temporal channels to the parietal cortex (C4‐Pz, T5‐P4). The standard deviation and mean value of the connection strength between the right and left temporal electrodes (T6‐T5) for the SZ and HC groups were 0.164 ± 0.10 and 0.182 ± 0.09, respectively. The significant difference between them was *p *= 0.034. As for functional connectivity features between other brain regions, SZ patients showed abnormally enhanced values ​​compared to HC subjects. Therefore, comparing the functional connectivity features at another level of significance for both groups is essential. Table 4 shows the statistical comparison between SZ patients and HC subjects using functional connectivity measurements based on PLI at a level of significance of *p*<0.001.

**TABLE 4:** The result of the statistical comparison between SZ patients and HCs according to significant differences at *p*<0.001

		Connectivity strength (mean ± std)							Connectivity strength (mean ± std)				
Chan.		SZ		HC		*p*	Chan.		SZ		HC		*p*
F7	F3	**0.494**	±0.09	0.466	±0.07	8.5e−05	C3	T4	0.305	±0.10	**0.336**	±0.09	0.00011
F7	F4	**0.351**	±0.10	0.315	±0.08	2.6e−06	C3	T5	0.172	±0.11	**0.211**	±0.08	5.1e−06
F7	T3	**0.105**	±0.07	0.082	±0.05	3.3e−05	C3	P3	0.143	±0.07	**0.168**	±0.09	0.00031
F7	C3	**0.287**	±0.10	0.230	±0.10	1.8e−10	C3	Pz	0.362	±0.09	**0.398**	±0.09	1.2e−05
F7	Cz	**0.210**	±0.09	0.160	±0.10	1.4e−09	C3	P4	0.445	±0.08	**0.506**	±0.08	2.5e−17
F7	P3	0.075	±0.05	**0.094**	±0.07	0.00029	C3	T6	0.161	±0.08	**0.205**	±0.08	4.0e−10
F7	Pz	0.141	±0.08	**0.169**	±0.09	0.00011	C3	O2	0.089	±0.07	**0.134**	±0.08	3.7e−11
F7	T6	**0.353**	±0.08	0.301	±0.10	7.4e−11	Cz	C4	**0.601**	±0.09	0.556	±0.10	2.2e−08
F7	O1	**0.253**	±0.07	0.302	±0.10	5.5e−10	Cz	P4	0.389	±0.09	**0.422**	±0.08	3.0e−05
F3	F8	0.255	±0.10	**0.284**	±0.09	0.00077	Cz	O2	0.077	±0.05	**0.115**	±0.08	2.3e−10
F3	C3	**0.098**	±0.07	0.076	±0.06	8.6e−05	C4	P3	0.176	±0.10	**0.231**	±0.10	9.3e−10
F3	T6	0.072	±0.05	**0.097**	±0.06	8.2e−07	C4	P4	0.192	±0.08	**0.222**	±0.09	8.0e−05
F4	F8	0.525	±0.10	**0.553**	±0.09	0.00097	C4	T6	0.375	±0.09	**0.435**	±0.09	2.2e−13
F4	T5	**0.130**	±0.08	0.095	±0.06	2.0e−07	C4	O2	0.090	±0.06	**0.123**	±0.07	1.3e−08
F4	P3	0.103	±0.06	**0.138**	±0.09	3.0e−07	T4	T5	**0.499**	±0.09	0.403	±0.09	3.3e−30
F4	T6	0.072	±0.05	**0.117**	±0.07	1.4e−15	T4	Pz	**0.121**	±0.09	0.089	±0.06	3.0e−06
F4	O1	**0.143**	±0.07	0.118	±0.08	0.00016	T4	P4	0.129	±0.09	**0.213**	±0.12	3.4e−18
F8	T3	**0.439**	±0.09	0.410	±0.09	0.00027	T5	P3	0.610	±0.09	**0.652**	±0.09	4.1e−07
F8	C4	0.127	±0.09	**0.169**	±0.13	1.2e−05	T5	Pz	0.384	±0.11	**0.423**	±0.09	1.6e−05
F8	P3	**0.105**	±0.08	0.083	±0.06	0.00052	T5	O1	0.421	±0.10	**0.493**	±0.10	3.3e−16
F8	Pz	**0.110**	±0.06	0.079	±0.05	1.6e−09	P3	P4	0.624	±0.08	**0.656**	±0.07	1.5e−06
F8	P4	0.068	±0.05	**0.101**	±0.07	5.1e−09	P3	O1	0.109	±0.07	**0.137**	±0.09	7.8e−05
T3	C3	**0.610**	±0.09	0.579	±0.10	0.00037	P3	O2	0.455	±0.11	**0.506**	±0.10	2.9e−08
T3	Cz	**0.355**	±0.11	0.321	±0.09	0.00015	Pz	T6	**0.283**	±0.10	0.230	±0.09	6.6e−10
T3	T4	0.138	±0.10	**0.178**	±0.08	2.4e−07	Pz	O2	0.436	±0.11	**0.470**	±0.09	7.4e−05
T3	T5	**0.167**	±0.09	0.130	±0.07	2.0e−07	P4	T6	**0.533**	±0.09	0.481	±0.09	3.1e−11
T3	Pz	**0.318**	±0.09	0.293	±0.08	0.00094	P4	O1	0.217	±0.13	**0.291**	±0.14	1.1e−10
T3	T6	0.127	±0.08	**0.158**	±0.07	2.8e−06	T6	O1	**0.422**	±0.08	0.381	±0.08	5.3e−09
T3	O1	0.140	±0.08	**0.169**	±0.09	9.3e−05	T6	O2	0.074	±0.05	**0.096**	±0.07	6.1e−05
C3	C4	0.557	±0.08	**0.581**	±0.07	0.00026	O1	O2	0.663	±0.12	**0.717**	±0.12	3.9e−07

*Note*: Bold fonts indicate higher connectivity strength.

Abbreviations: HC, Healthy control; *p*, *p‐*value; SZ, Sczofrenia.

**FIGURE 8:**
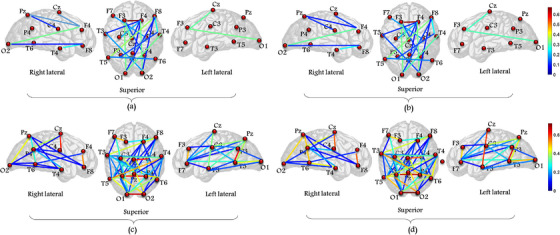
Comparison of functional connectivity strength of SZ patients ((a) and (c)) and HC subjects ((b) and (d)). The first row is the brain visual representation of the group averaged critical connections based on 0.001⩽*p*<0.05. The second row compares the group average functional connectivity between SZ patients and HC subjects at *p*<0.001. The color bar indicates the connectivity strength.

Table 4 revealed that the connectivity strengths between different brain regions were commonly weaker in SZ patients than in HC subjects. More specifically, the average connectivity strengths between the nodes in the parietal cortex and those in the central, occipital, and temporal regions were generally weaker in SZ patients than in HC subjects. For the exemplary electrode pairs such as T4‐P4, P3‐T5, P4‐C3, P3‐C4, P3‐O1, P3‐O2, and P4‐O1, the average beta functional connectivity strengths with standard deviation were 0.129 ± 0.09, 0.610 ± 0.09, 0.445 ± 0.018, 0.176 ± 0.10, 0.109 ± 0.07, 0.455 ± 0.11, and 0.217 ± 0.13 for SZ patients as well as 0.213 ± 0.12, 0.652 ± 0.09, 0.506 ± 0.08, 0.231 ± 0.10, 0.137 ± 0.09, 0.506 ± 0.10, and 0.291 ± 0.14 for HC subjects, respectively. The *p*‐values, the degree of the statistically significant differences between both groups, were 3.4e−18, 4.1e−07, 2.5e−17, 9.3e−10, 7.8e−05, 2.9e−08, and 1.1e−10 for these channel pairs, respectively. The partial lack of spatial details on the group average PLI functional connectivity strength for both levels of significance restricts the interpretation of the effects of SZ on the brain. Visual presentation of connectivity strengths between brain regions may be more useful. Figure 8 visually summarizes the functional connectivity strengths and significant differences of SZ patients and HC subjects.

The beta functional connectivity dynamics revealed that SZ patients had much weaker short‐range connections between the parietal and the occipital channels than HC subjects. Figure 8 shows that the significant differences between the two groups were reduced for long‐range functional connections. For both groups, it has been reported that there is a high flow of information on short‐range connections in general. As the spatial range between channels increased, the statistically significant difference between the SZ and HC groups decreased.

## 4 DISCUSSION

The present study addressed the challenge of discriminating SZ abnormalities from HC subjects using resting‐state EEGs. In this context, a DL‐based automated SZ diagnosis pipeline has been proposed. In order to exploit the superior capabilities of DL algorithms on image processing,^22^, ^37^, ^41^ the investigation for an efficient EEG representation approach was focused. The results proved that brain functional connectivity dynamics based on PLI are a robust representation technique to discriminate SZ patients from HC subjects with satisfactory classification accuracy. Furthermore, compared to other subband datasets, a much higher classification accuracy of 96.02% was achieved using the FCR inputs of beta band.

Clinical literature has emphasized the importance of the role of especially high‐frequency oscillatory activity in the functional connectivity phenomenon.^42^ This phenomenon can reflect the interaction between different brain regions underlying higher neural behavior and possibly even consciousness.^42^, ^43^ An essential hallmark of SZ is impaired brain connectivity and significant decreases in perception networks.^43^, ^44^, ^45^, ^46^ More specifically, disruption of functional connectivity for high‐frequency beta‐rhythm is considered a fundamental feature of SZ.^42^ This study supports the clinical literature due to the best discrimination performance for the beta band of the proposed model.

In order to enhance the existing accuracy performance in SZ diagnosis, this paper explored deeper architectures. However, deeper neural networks are more challenging to train due to the problems of vanishing and exploding gradients.^27^, ^37^, ^38^ Depending upon the number of layers, several variants of ResNet were used to overcome this issue, including ResNet‐18, ResNet‐50, and ResNet‐101. Among these variants, ResNet‐50 improved the accuracy of SZ diagnosis by around 3% with its deeper architecture compared to ResNet‐18. For the ResNet‐50, the results demonstrated that the residual network methodology works effectively. However, the ResNet‐101 model failed to achieve accuracy performance from considerably increased depth. The decrease in classification accuracy for ResNet‐101 can be associated with the low complexity of the data set. Also, Resnet‐101 is more prone to overfitting due to its larger architecture.

After statistical analysis of the average connectivity strength between different brain regions for the beta band, significant differences (*p* < 0.05) were reported between SZ and HC for most EEG channel pairs. Furthermore, the results demonstrated that SZ causes a decrease and distributed inefficiency of cortical communication in patients. The statistical results revealed that SZ affects functional connections associated with many cognitive tasks, such as attention, working memory, and executive function.

### 4.1 Comparison with prior work

This section compared the proposed pipeline to remarkable state‐of‐the‐art studies. Classification accuracy and other details of previous studies are systematically summarized in Table 5. As shown in Table 5, the proposed model provided satisfactory performance for SZ diagnosis. Due to the different data sets, data splitting rates in the training phase, window sizes for segmentation, and x‐fold cross‐validation, the comparison results do not fully reflect the superiority of the proposed models over each other. However, it can give a reliable and binding idea of the robustness of the state‐of‐the‐art models. For example, Calhas et al.^30^ performed validation based on leave‐one‐subject‐out cross‐validation. However, this study created a data pool of FCR images of healthy subjects and SZ patients before cross‐validation. In the study that inspired this work, Phang et al.^24^ selected a window size of 1 s for dynamic connectivity patterns in EEGs. Nonetheless, the effectiveness of the proposed model becomes apparent, especially when the previous works using the same data set are considered.^9^, ^24^, ^30^, ^34^ The satisfactory classification accuracy in this study is mainly due to the use of FCR input and residual neural networks. The FCR input based on PLI provided valuable information about the complex relationships between different brain regions in a multidimensional way. Then, the fact that the ResNet model extracted deeper and more robust features directly increased accuracy.

**TABLE 5:** Related work overview for SZ diagnosis and comparison of resulting classification performances

Author(s)	Channels	EEG task	Participants	Feature extraction	Input formulation	Classifier	Accuracy
Kim et al.^16^	19	Resting state	90 SZ patients 90 HC subjects	Spectral band power based fast Fourier transform	—	ROC analysis	62.2%
Buettner et al.^17^	19	Resting state	14 SZ patients 14 HC subjects	Spectral analysis of frequency bands	—	RF	71.43%
Piryatinska et al.^9^	16	Resting state	45 SZ patients 39 HC subjects	ε‐complexity coefficients	—	RF SVM	85.3% 81.07%
Dvey‐Aharon et al.^19^	64	Visual stimuli P300 task	25 SZ patients 25 HC subjects	Time‐frequency transformation and feature‐optimization	—	Cross‐validation based on ‘leave one out’	93.9%
Santos‐Mayo et al^20^	17	Auditory oddball task	31 SZ patients 16 HC subjects	Time‐frequency domain features	—	SVM MLP	92.23% 93.42%
Phang et al.^24^	16	Resting state	45 SZ patients 39 HC subjects	Automatically	2D time and frequency domain connectivity representation	Multi‐domain connectome CNN model	93.06%
Oh et al^6^	19	Resting state	14 SZ patients 14 HC subjects	Automatically	Time domain representation (channel × length)	11‐layered deep CNN model	98.07%
Shalbaf et al.^25^	19	Resting state	14 SZ patients 14 HC subjects	Automatically	Time‐frequency representation (scalogram images)	ResNet‐18+SVM	98.60%
Naira et al.^34^	16	Resting state	45 SZ patients 39 HC subjects	Automatically	Correlation matrices representation	A basic CNN pipeline	90%
Calhas et al.^30^	16	Resting state	45 SZ patients 39 HC subjects	Automatically	Time‐frequency representation	Siamese neural network architecture (SNN)	95%
Present work	16	Resting state	45 SZ patients 39 HC subjects	Automatically	Brain functional connectivity representation based on phase lag index	ResNet‐50	96.02%

*Note*: The table categorizes the related work in terms of EEG properties, feature extraction technique, input formulation, and classifier types.

### 4.2 Limitations and disadvantages

Although the proposed FCR‐based ResNet framework has shown satisfactory performance, the majority of current automated SZ diagnosis approaches still have some remarkable limitations. The robustness of the DL‐based diagnosis framework depends on the availability of large datasets. However, the creation of large medical datasets is a big challenge. Thus, this study suffered from the limited data. Using large‐scale data instead of limited data would have increased the performance of the proposed model and allowed for more reliable results. Another issue is the lack of long‐term EEG monitoring records, especially for SZ patients. More specifically, the absence of both patient and healthy EEG trials for the same individual makes patient‐specific analyzes impossible.

## 5 CONCLUSION

This paper revealed that it is achievable to effectively discriminate SZ patients from HC subjects by combining EEG functional connectivity patterns and ResNet variants. The excellent results demonstrated that the topographical representations of symmetrical connectivity matrices provide a satisfactory level of input quality to the ResNet model. İt is crucial not only to provide an automated diagnosis system but also to provide potential biomarkers for clinical use. In this context, brain connectivity dynamics in the beta band were suggested as the robust biomarker for automated SZ diagnosis. Furthermore, the inference that SZ causes a decrease in cortical communication can be considered another valuable biomarker.

In the future, it is planned to investigate the effectiveness of the proposed model in detecting additional types of mental disorders or illnesses. Future work can also focus on improving the current performance by applying modifications to the ResNet model based on dilated convolution filter.

## CONFLICT OF INTEREST

The author has no competing interests to declare that are relevant to the content of this article.

## ETHICS STATEMENT

The dataset used in this work is openly accessible and free to the public. This work has no direct interaction with a human or animal entity.

## Data Availability

The data set used in this article is publicly available and can be downloaded from: http://brain.bio.msu.ru/eegschizophrenia.htm
